# Endocannabinoids Produced by White Adipose Tissue Modulate Lipolysis in Lean but Not in Obese Rodent and Human

**DOI:** 10.3389/fendo.2021.716431

**Published:** 2021-08-09

**Authors:** Chloé Buch, Tania Muller, Julia Leemput, Patricia Passilly-Degrace, Pablo Ortega-Deballon, Jean-Paul Pais de Barros, Bruno Vergès, Tony Jourdan, Laurent Demizieux, Pascal Degrace

**Affiliations:** ^1^Team Pathophysiology of Dyslipidemia, INSERM UMR1231, Université de Bourgogne Franche-Comté, Dijon, France; ^2^Department of Digestive, Thoracic and Surgical Oncology, University Hospital, Dijon, France; ^3^Lipidomic Platform, INSERM UMR1231, Université de Bourgogne Franche-Comté, Dijon, France; ^4^Department of Endocrinology-Diabetology, University Hospital, Dijon, France

**Keywords:** endocannabinoid system (ECS), adipose tissue, lipolysis and fatty acid metabolism, obesity, cannabinoid (CB) receptor 1, cAMP, Akt, hormono-sensible lipase

## Abstract

White adipose tissue (WAT) possesses the endocannabinoid system (ECS) machinery and produces the two major endocannabinoids (ECs), arachidonoylethanolamide (AEA) and 2-arachidonoylglycerol (2-AG). Accumulating evidence indicates that WAT cannabinoid 1 receptors (CB1R) are involved in the regulation of fat storage, tissue remodeling and secretory functions but their role in controlling lipid mobilization is unclear. In the present study, we used different strategies to acutely increase ECS activity in WAT and tested the consequences on glycerol production as a marker of lipolysis. Treating lean mice or rat WAT explants with JLZ195, which inhibits ECs degrading enzymes, induced an increase in 2-AG tissue contents that was associated with a CB1R-dependent decrease in lipolysis. Direct treatment of rat WAT explants with AEA also inhibited glycerol production while mechanistic studies revealed it could result from the stimulation of Akt-signaling pathway. Interestingly, AEA treatment decreased lipolysis both in visceral and subcutaneous WAT collected on lean subjects suggesting that ECS also reduces fat store mobilization in Human. In obese mice, WAT content and secretion rate of ECs were higher than in control while glycerol production was reduced suggesting that over-produced ECs may inhibit lipolysis activating local CB1R. Strikingly, our data also reveal that acute CB1R blockade with Rimonabant did not modify lipolysis *in vitro* in obese mice and human explants nor *in vivo* in obese mice. Taken together, these data provide physiological evidence that activation of ECS in WAT, by limiting fat mobilization, may participate in the progressive tissue remodeling that could finally lead to organ dysfunction. The present findings also indicate that acute CB1R blockade is inefficient in regulating lipolysis in obese WAT and raise the possibility of an alteration of CB1R signaling in conditions of obesity.

## Introduction

The role of peripheral endocannabinoid system (ECS) in regulating energy homeostasis has been the focus of many studies and strong evidence suggests that cannabinoid 1 receptors (CB1R) activation by the two major endocannabinoids (ECs), N-arachidonoylethanolamide (also referred to as anandamide or AEA) and 2-arachidonoylglycerol (2-AG), stimulates food intake and exerts coordinated actions on several organs finally promoting energy storage and fat accumulation (1). The existence of the ECS machinery in white adipose tissue (WAT) (2–5) and the fact that adipocyte-specific CB1R knock-out mice remain leaner than their wild-type littermates when fed a high fat diet support the view that CB1R regulates adipocyte biology, including lipid homeostasis (6, 7). Regulation of WAT mass is a dynamic process together with lipid accumulation and mobilization. During fasting conditions, lipolysis is activated leading to the release NEFA and glycerol from triglyceride stores into the circulation. Catecholamines and insulin are two major regulators of this tightly controlled metabolic pathway. Catecholamine binding to beta-adrenergic receptors stimulates the adenylyl cyclase/cAMP/protein kinase A (PKA) cascade ultimately activating perilipin and hormone sensitive lipase (HSL) (8). During the fed state, lipolysis is negatively regulated by insulin which triggers cAMP breakdown by phosphodiesterase 3B (PDE3B), through the activation of phosphatidylinositol 3-kinase (PI3K) and Akt dependent pathways (9, 10). It should be noted that insulin is also involved in regulating lipolysis in the fasting state since this hormone exerts an antilipolytic action even at relatively low plasma concentrations (11).

Interestingly, obesity development in mice and humans is associated with an increase in circulating ECs (2, 12, 13) suggesting that fat depots are an important source for plasma ECs. Although their capacity to induce physiological and pathophysiological exocrine effects on extra adipose organs is still matter of debate (14), data support that locally produced ECs are involved in autocrine regulation processes for WAT homeostasis. Thus, it has been demonstrated that CB1R activation in adipocytes favors energy storage by different additional mechanisms, such as enhancement of lipoprotein lipase activity, glucose uptake and adipogenesis (4, 6, 15–17). Numerous studies have reported an ECS involvement in the regulation of fat mobilization (18, 19). However, conclusions were mostly drawn from analysis of gene or protein expression obtained from animals that were chronically treated with CB1R antagonists (20, 21). Thus, these observations might be the result of indirect responses. Moreover, studies showing direct evidence of ECS-induced lipolysis modulation through physiological approaches are very scarce and often contradictory. For instance, some *in vitro* experiments reported either an inhibitory or a stimulating effect of ECs on lipolytic activity likely depending on the presence of insulin (22–24).

The present study was designed to clarify the direct effects of an acute activation of CB1R on adipose tissue lipolysis and precise the mechanisms involved. This notion deserves particular attention since a decrease in fasting lipolysis is predicted to promote fat storage and therefore to contribute to the metabolic deregulations associated with WAT remodeling (25, 26). Using both *in vivo* and *in vitro* approaches, we provide physiological evidence that acute CB1R activation in WAT reduces fasting lipolysis both in rodent and human. In addition, mechanistic analysis strongly supports it could result from the stimulation of PI3K/Akt signaling pathway. Strikingly, we also show that blockade of CB1R does not modify lipolysis in obese WAT while ECs production is increased.

## Materials and Methods

### Animals and Diets

C57BL/6J male mice (10-12 week-old) and Wistar rats (250-350 g) from JanvierLabs (Le Genest Saint Isle, France) were housed on a 12/12h light/dark schedule at 22–23°C with *ad libitum* access to water and to a standard diet (STD; A04; UAR, Epinay-sur-Orge, France). For experiments using high fat diet (HFD) mice, 4-week-old C57BL/6J mice were fed *ad libitum* a diet containing 60% of calories from lipids (lard), 20% from protein and 20% from carbohydrates (cat. no. E15742-34, Ssniff, Soest, Germany) for 16 weeks. In the meantime, control mice (LEAN) were fed the corresponding low fat diet containing 5% of calories from lard (cat. no. S9259-E010, Ssniff). Animals were fasted before each *in vivo* experiment or tissue collection.

### Drugs

All chemicals and media were supplied by Sigma (Saint-Quentin-Fallavier, France) except for Rimonabant (SR141716) purchased from Sanofi Aventis (Paris, France); AEA and JZL195 from Bio-Techne (Noyal Châtillon sur Seiche, France); JD-5037 from CliniSciences (Nanterre, France); Arachidonoyl Ethanolamide-D4 and Forskolin from Interchim (Montluçon, France).

All drugs administrated by intraperitoneal (i.p.) injection were diluted in 0.1% DMSO, 0.025% TWEEN 80, 0.9% NaCl or simply dissolved in DMSO when added in the culture medium.

### *In Vivo* Experiments With Lean and Obese Mice

#### JZL195 experiments

24h-fasted mice were subjected to an intraperitoneal injection of the dual inhibitor of the degradation enzymes for AEA and 2-AG, FAAH, and MAGL, JZL195 (20 mg/kg) or vehicle (0.1% DMSO/0.025% Tween 80 in 0.9% NaCl). Blood samples were recovered every hour at the tail tip and for 3 hours. Blood samples were centrifuged 10 min at 6,500 g 4°C and plasma was recovered in order to measure glycerol content by using colorimetric glycerol FS assays (DiaSys, Condom, France). At the end of 3 hours, mice were sacrificed and epididymal WAT was recovered for endocannabinoid quantitation and determination of glycerol release by culture explants as described for rat explants.

#### Experiments on HFD mice

Basal lipolysis was evaluated in 24h-fasted obese mice subjected to an intraperitoneal injection of Rimonabant (10 mg/kg) or JD5037 (3 mg/kg) or vehicle (0.1% DMSO/0.025% Tween 80 in NaCl 0.9%). Blood samples were collected at 0, 15, 30, 45 and 60 min for determination of plasma glycerol content. Stimulated lipolysis was measured similarly after i.p. administration of BRL37344 (5 mg/kg). To study the impact of the specific CB1R inactivation on Akt activity and cAMP WAT levels, some mice were injected with Rimonabant and sacrificed at 15 and 45 min respectively. Some animals were dedicated to the preparation of WAT explants for glycerol and ECs production.

### Preparation and Treatment of Rodent and Human WAT Explants

#### Rat and mice epididymal explants

After collection, epididymal WAT was rinsed in warm medium (DMEM-HAM/F12) supplemented with 1% BSA and 0,12% sodium bicarbonate and dissected into ~10 mg pieces. Then, 5−6 explants were pre-incubated 30 min in a 5% CO2 atmosphere at 37°C under slight agitation in the same medium.

For JZL195 experiments, tissue ECs content were measured after a 3h-incubation of explants with 10 µM of the inhibitor or vehicle (0,1% DMSO). Some JZL195-treated explants were also rinsed and placed in fresh DMEM-HAM/F12 for 1 h in order to determine glycerol and/or NEFA production in presence or not of norepinephrine (1 µM) and Rimonabant (1 µM).

In other experiments, we directly tested the effect of AEA (5 µM) on NE-stimulated glycerol production by rat WAT explants in the presence or not of Rimonabant (1 µM).

For experiments dedicated to signaling pathway analyses, WAT explants were treated with either forskolin (10 µM) and AEA (5 µM) or vehicle (0,1% DMSO) during 15 min for cAMP assays or either with AEA (5 µM) or vehicle alone during 15 min for immunoblotting assays. At the end of incubation time, explants were collected and frozen in liquid nitrogen for further analyses.

#### Human visceral and subcutaneous WAT explants

Abdominal visceral and subcutaneous adipose tissue (VAT and SAT) was collected from adult male patients who underwent abdominal surgery at the University Hospital (Dijon, France). The participants were selected based on their body mass index (BMI) calculated as mass/height^2^ and categorized as normal-weighted (BMI<29.9 kg/m^2^) and obese (BMI>29.9 kg/m^2^). Ten normal-weighted (BMI: 24.5 ± 0.55; age: 49 ± 3 years) and 10 obese (BMI: 38.3 ± 0.95; age: 44 ± 4) individuals were recruited. Patients with inflammatory bowel disease, cancer, infection, previous bariatric surgical procedures or active prescription for medication that could affect lipolysis were excluded from the study. Once recovered, tissues were kept in DMEM-HAM/F12 supplemented with 1% BSA and 0,12% sodium bicarbonate and immediately transferred to the laboratory. Then tissues were submitted to the same experimental protocol as rodent explants.

### Glycerol, NEFA, and cAMP Assays

Glycerol and NEFA in plasma or culture medium were measured by colorimetric method using respectively glycerol FS and NEFA FS commercial kits (DiaSys, Grabels, France) according to supplier’s information. Intracellular cAMP levels were determined by a competitive ELISA method using a cAMP Complete ELISA kit (Enzo Life Science, Villeurbanne, France). Briefly, frozen WAT samples were extracted with 0.1 M of HCl in a Mini-Beadbeater (BioSpec Products) and then centrifuged twice at 800 g for 10 min at 4°C. The cAMP assay was performed on the supernatant following the supplier’s instructions.

### Endocannabinoid Extraction and Quantitation by Mass Spectrometry

Extraction procedure was adapted from (27). Briefly, adipose tissue samples (50-80 mg) were homogenized in a Mini-Beadbeater (BioSpec Products, Bartlesville, OK, United states) with 500 µL of ice-cold MeOH/Tris (50 mM, pH 8) 1:1 containing 11.2 ng of d4-anandamide standard. Then, 2 ml of ice-cold CHCl3/MeOH 1/1 and 500 µL of Tris (50 mM, pH 8) were added to each homogenate. Tubes were vortexed and centrifuged for 10 min at 3,000 g (-2°C). The organic layer was recovered, and the water phase was extracted one more time with ice-cold CHCl_3_. The combined extracts were dried under N_2_ and resolubilized in 120 µL of ice-cold CHCl_3_. Proteins were precipitated with 2 ml of ice-cold acetone, incubated for 30 min at -20°C, and centrifuged for 10 min at 3,000 g (-2°C). The supernatant was carefully transferred to new tubes and dried under N_2_.

ECs extraction from sample medium was performed according to (28). Briefly, Supelclean™ LC-18 SPE cartridges (Sigma, Saint-Quentin-Fallavier, France) were conditioned with 1 ml acetonitrile followed by 1 ml 0.1M phosphate buffer (pH 9.1). Then 1ml of sample medium added with 2 ng of d4-anandamide standard and acidified with 20 µl ortho-phosphoric acid (85%) was loaded onto the cartridge. Cartridges were washed with 1ml 40% acetonitrile and dried by centrifugation. Elution was performed with 1ml acetonitrile/ammonia $\left(\text{N}{\text{H}}_{4}^{+}\right)$ (98:2, v/v) and dried under N_2._ Finally, the dried residue was reconstituted in MeOH and injected into a 1200 LC system coupled to a 6460-QqQ MS/MS system equipped with an electrospray ionization source (Agilent Technologies) as previously described (22). ECs were quantitated by the isotope dilution method by using deuterated standards.

### Western Blotting

For immunoblotting, WAT proteins were extracted using a Mini-Beadbeater (BioSpec Products) in RIPA lysis buffer (NaCl 150 mM, Triton 1%, sodium deoxycholate 0.5%, SDS 1%, Tris-HCl 50 mM, pH 8) supplemented with a phosphatase and protease inhibitor cocktail. Then, extracts were incubated on ice for 1 h and centrifuged twice at 15,000 g for 20 min at 4°C to clarify protein lysate from fat. Protein concentration was determined with the BCA Protein Assay Kit (Sigma, Saint-Quentin Fallavier, France). 25 µg of proteins were resolved in gradient (4–20%) SDS-PAGE gels (Bio-Rad, Marnes-la-Coquette, France) and blotted onto nitrocellulose membranes with the Transblot system (Bio-Rad). After 2 h in blocking buffer (BSA 5%), the membranes were incubated with primary antibodies recognizing phospho-Akt T308, phospho-Akt S473, phospho-Akt S474, phospho-PKA-C T197, phospho-HSL S660, total form of Akt, PKA-C and HSL, and β-actin (**Table S1**). Appropriate secondary antibodies conjugated to horseradish peroxidase were used for detection. Finally, proteins of interest were visualized by enhanced chemiluminescence with the Bio-Rad Chemi-Doc MP Imaging System after substrate incubation (Clarity, Bio-Rad). Bands were quantified by densitometric analysis performed by ImageLab software. All antibodies were purchased from Cell Signaling (Danvers, MA, United States) except β-actin (Merck-Millipore, Molsheim, France).

### RT-PCR

Total mRNAs from tissues were extracted with Tri-Reagent (Euromedex, Souffelweyersheim, France), and 1 µg RNA was reverse transcripted with the Iscript cDNA Kit (Bio-Rad). Real- time PCR was performed as described previously (22) by using a StepOnePlus real-time PCR system (Life Technologies, Saint-Aubin, France). For each gene, a standard curve was established from four cDNA dilutions (1/5 to 1/100) and used to determine the relative gene expression variation after normalization with the geometric mean of three housekeeping genes, TATA box binding protein (TBP), L38, and ATP5e. Forward and reverse primer sequences used for amplification are presented in **Table S2**.

### Statistical Analysis

Results are expressed as means ± SEM. Data were analyzed statistically with GraphPad InStat software (GraphPad Software, La Jolla, CA) by two-way ANOVA followed by the Tukey *post hoc* test or by Student’s *t-*test. When appropriate, data were analyzed by paired sample t-test. Differences were considered significant at *P*<0.05.

## Results

### Elevation of Endocannabinoid Tone by JZL195 Decreases Fasting Lipolysis in WAT

**Figure 1:**
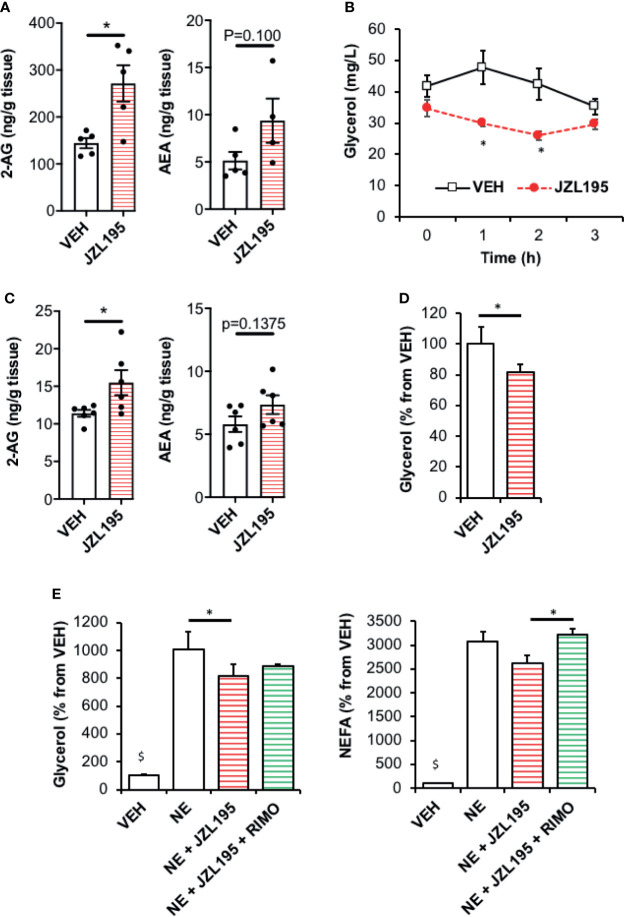
Elevation of endocannabinoid tone in WAT by JZL195 decreases lipolysis. **(A)** 2-AG and AEA levels in epididymal WAT collected from mice 3 hours after an intraperitoneal injection of vehicle (VEH) or JZL195 (20 mg/kg); n=5/group. **(B)** Kinetic of plasma glycerol (0, 1h, 2h and 3h) in mice after an intraperitoneal injection of VEH or JZL195 (20 mg/kg); n=5/group. **(C)** 2-AG and AEA levels measured in rat WAT explants after a 3-hour incubation with VEH or JZL195 (10 µM); n=6/group. **(D)** Basal glycerol production by WAT explants treated 3 hours with VEH or JZL195 (10 µM). Explants were treated in triplicate and experiments repeated on 3 different rats (n=3/group). **(E)** Glycerol and non-esterified fatty acids (NEFA) production by rat WAT explants pre-incubated 3 hours with VEH or JZL195 (10 µM) and treated 1 additional hour with VEH, JZL195 or JZL195+Rimonabant (RIMO, 1 µM) in the presence of norepinephrine (NE, 1 µM). Explants were treated in triplicate and experiments repeated on 3 different rats (n=3/group). ^$^p < 0.001 VEH *vs* other treatments. Results are expressed as mean ± SEM. *p < 0.05.

We designed a set of *in vivo* and *in vitro* experiments using JZL195, a dual inhibitor of the ECs degrading enzymes, fatty acid amide hydrolase (FAAH) and monoacylglycerol lipase (MAGL) as a strategy to elevate WAT ECS tone. First, JZL195 treatment induced an increase in 2-AG levels in WAT collected three hours after the injection in comparison with vehicle-treated control mice while the same trend was observed for anandamide (p=0.100) (**Figure 1A**). Interestingly, the JZL195 injection was followed by a significant decrease in plasma glycerol levels at time 1 h and 2 h that was not observed in control mice suggesting that the activation of ECS was associated with a decrease in fat mobilization (**Figure 1B**).

Because this *in vivo* JZL195 treatment could have impacted lipolysis by modifying the ECS tone in extra adipose tissues and/or altering norepinephrine production by sympathetic nerve endings, we designed an *ex-vivo* experiment in order to selectively test the effects of local ECS activation on lipolysis. For this, rat WAT explants were directly treated with JZL195 for three hours before measuring glycerol and FFA production rates. In these conditions, JZL195 treatment also increased 2-AG tissue content (**Figure 1C**) and was still associated with a decrease in both basal (**Figure 1D**) and catecholamine-stimulated lipolysis (**Figure 1E**). Interestingly, blockade of CB1R with Rimonabant in explants treated with JZL195 normalized glycerol and NEFA production (**Figure 1E**).

### CB1R Activation Decreases Lipolysis in Rat WAT Explants Through an Akt-Dependent Mechanism

**Figure 2:**
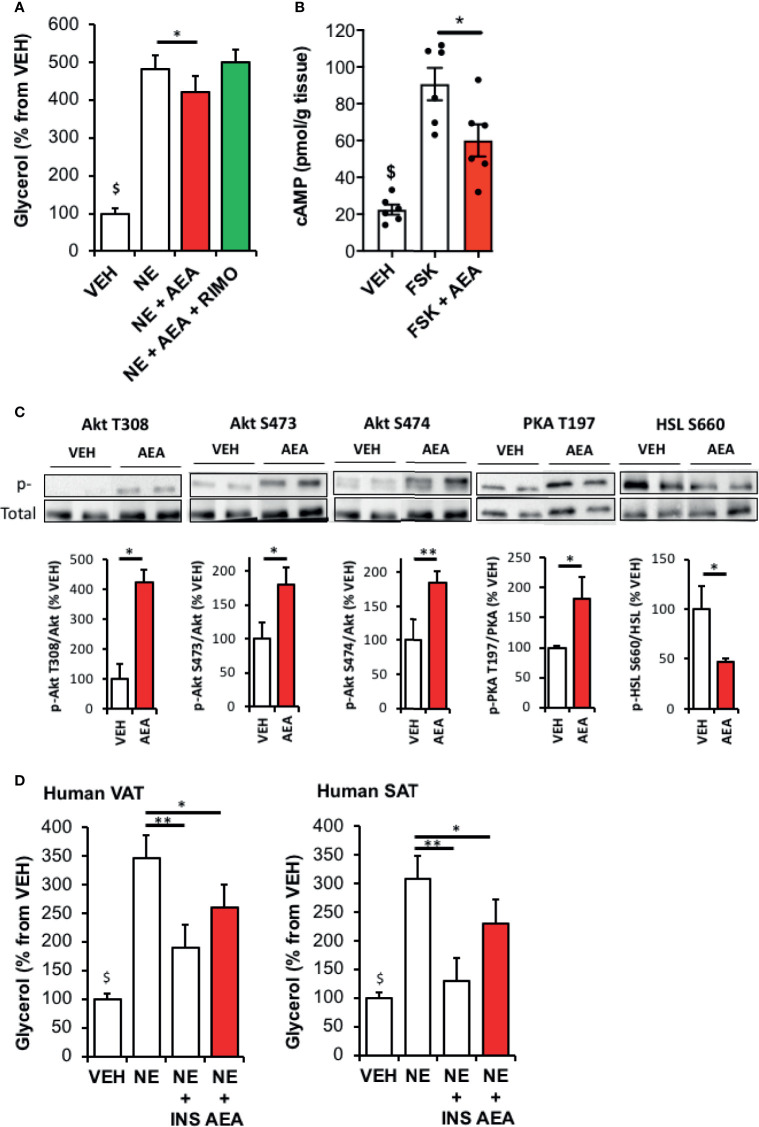
CB1R activation by AEA decreases WAT lipolysis through an Akt-dependent mechanism. **(A)** Glycerol production by rat WAT explants pre-incubated 30 min with vehicle (VEH) or AEA (5 µM) and incubated 1 hour with VEH, AEA, AEA+Rimonabant (RIMO, 1 µM) in the presence of norepinephrine (NE, 1 µM). Explants were treated in triplicate and experiments repeated on 3 different rats (n=3/group). ^$^p < 0.001 VEH *vs* other treatments. **(B)** Intracellular cAMP content in rat WAT explants treated 15 min either with VEH, forskolin (FSK, 10 µM) or FSK+AEA (5 µM). Explants were treated in triplicate and experiments repeated on 3 different rats (n=3/group). ^$^p < 0.001 VEH *vs* other treatments. **(C)** Representative immunoblots and densitometry analyses of the phosphorylation level of Akt (T308, S473, S474), PKA-C (T197) and HSL (S660) in rat WAT explants treated 15 min with VEH or AEA (5 µM). **(D)** Glycerol production by Human TAV or SAT explants pre-incubated 30 min with VEH or AEA (5 µM) and incubated 1 hour with VEH, AEA or insulin (INS, 500 nM) in the presence of norepinephrine (NE, 1 µM). Explants were treated in triplicate and experiments repeated on 7 different subjects (n=7/group). ^$^p < 0.001 VEH *vs* NE and NE+AEA. Results are expressed as mean ± SEM. *p < 0.05; **p < 0.01.

The model of rat WAT explants treated with AEA was further used to study how CB1R signaling may alter lipolysis. We first validated our model showing that compared with vehicle, norepinephrine-stimulated glycerol release was decreased in explants treated with AEA in a CB1R-dependent manner as this effect was abrogated by co-treatment with Rimonabant (**Figure 2A**) and did not exist in CB1R KO mice (**Figure S1**).

In another set of experiments, we observed that the stimulating effect of forskolin (direct activator of adenylyl cyclase) on intracellular cAMP levels was counteracted in the presence of AEA (**Figure 2B**). This indicate that, in this model of WAT explants, CB1R is able to modulate cAMP levels independently of beta-adrenergic receptors signaling but rather through inhibiting adenylyl cyclase and/or stimulating PDE-3B activity. Consistent with this, Akt (T308/S473 and S474) and PKA T197 phosphorylation was stimulated by AEA treatment and associated with a decrease in HSL activation (**Figure 2C**).

In order to verify whether our findings also apply to Human, we studied the impact of AEA on norepinephrine-stimulated glycerol production by VAT and SAT collected on lean subjects.

As expected, glycerol release in medium was increased by norepinephrine and decreased by insulin for both fat depots. Interestingly, AEA treatment decreased norepinephrine-stimulated glycerol release suggesting ECS also reduced the capacity to mobilize fat stores in human WAT (**Figure 2D**).

### AEA Production Is Increased in WAT of HFD Mice While Lipolysis Is Reduced

**Figure 3:**
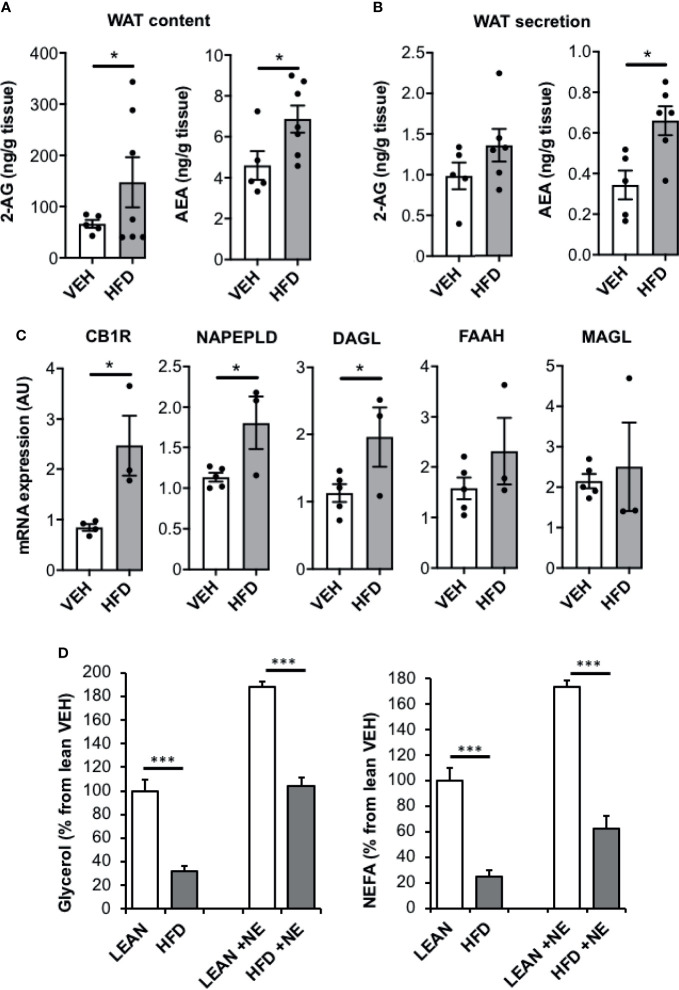
AEA production is increased in WAT of HFD mice while lipolysis is reduced. **(A)** 2-AG and AEA levels in epididymal WAT of mice fed with a high fat diet (HFD) compared to standard diet (LEAN); n=5/group. **(B)** Production of 2-AG and AEA by WAT explants from HFD and LEAN mice after 2 hours of incubation in culture medium added with JZL195 (10 µM) to limit the degradation of ECs secreted over time (n=5/group). **(C)** mRNA expression of CB1R, NAPEPLD, DAGL, FAAH and MAGL in epididymal WAT of LEAN and HFD mice (n=5/group). **(D)** Glycerol and nonesterified fatty acid (NEFA) production by WAT explants from HFD and LEAN mice after 1 hour of incubation in the presence of norepinephrine stimulation (NE, 1 µM) or not. Explants were treated in triplicate and experiments repeated on 3 different mice (n=3/group). Results are expressed as mean ± SEM. *p < 0.05; ***p < 0.001.

To test whether the ECS also regulate fat mobilization in obese WAT, we challenged mice with a HFD for 16 weeks. Compared to lean mice, HFD mice displayed hyperinsulinemia and global insulin-resistance as illustrated by insulin tolerance test and p-Akt levels in adipose tissue (**Figure S2**). Interestingly and as expected, epididymal fat pads of HFD mice had significant higher tissue contents of 2-AG and AEA compared to lean mice (**Figure 3A**). We further measured the secretion of ECs in the culture medium of freshly collected epididymal tissue explants from HFD and lean mice. In accordance with tissue contents, AEA levels produced for 2h were higher in epididymal HFD explants than in control while 2-AG levels were not significantly increased (**Figure 3B**). Consistent with an increase in ECS tone in the WAT of obese mice, mRNA levels of genes coding for CB1R, NAPEPLD and DAGL were also higher than in control while that coding for the ECs degrading enzymes FAAH and MAGL, were unchanged (**Figure 3C**). Interestingly, we concomitantly observed that glycerol and NEFA release by fresh WAT derived from HFD mice was lower compared with control mice both in basal conditions and in response to norepinephrine stimulation (**Figure 3D**). On the whole, it can be assumed that ECs over-produced in obese WAT locally activate CB1R and exert the same inhibitory effect on lipolysis described earlier in settings using exogenous AEA.

### Blockade of CB1R Does Not Modify Lipolysis in Obese Mice and Human WAT

**Figure 4:**
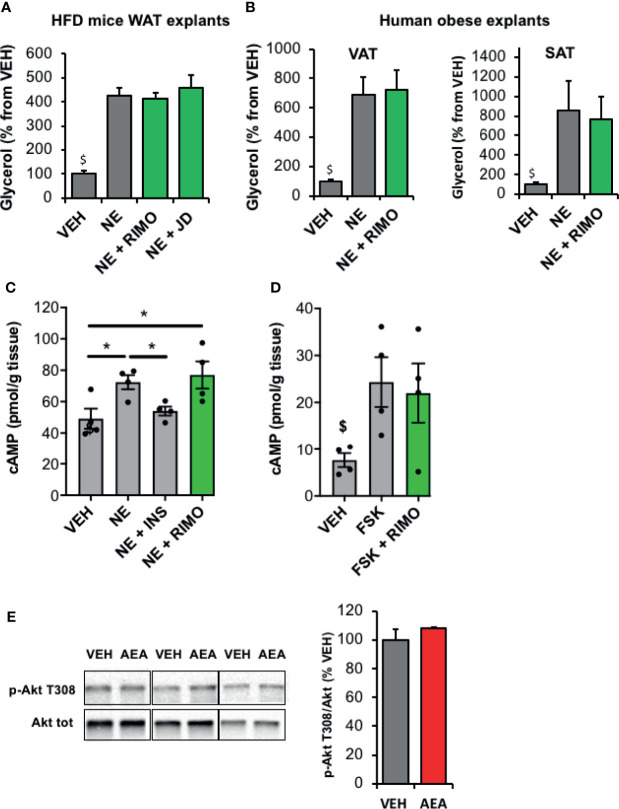
Blockade of WAT CB1R does not modify lipolysis in obese mice and human *in vitro*. **(A)** Glycerol production by WAT explants from mice fed with a high fat diet (HFD) and treated 1 hour with vehicle (VEH), Rimonabant (RIMO, 1 µM) or JD5037 (0.3µM) in the presence of norepinephrine (NE, 1 µM). Explants were treated in triplicate and experiments repeated with 3 different HFD mice (n=3/group). ^$^p < 0.001 VEH *vs* other treatments. **(B)** Glycerol production by abdominal VAT or SAT explants from obese subjects measured in the medium after 1-hour treatment with VEH or RIMO (1 µM) in the presence of NE (1 µM). Explants were treated in triplicate and experiments repeated with tissue from 6 different obese subjects (n=6/group). ^$^p < 0.001 VEH *vs* other treatments. **(C)** Intracellular cAMP content measured in WAT explants from HFD mice treated 1 hour with VEH, insulin (INS, 1 nM) or RIMO (1 µM) in the presence of NE (1 µM); n=4/group. **(D)** Intracellular cAMP content measured in WAT explants from HFD mice treated 15 min with VEH, forskolin (FSK, 10 µM) or FSK + RIMO (1 µM); n=5/group. ^$^p < 0.001 VEH *vs* other treatments. **(E)** Representative immunoblots and densitometry analyses of the phosphorylation level of Akt (T308) in WAT explants collected from HFD mice and treated 20 min with VEH or AEA (5 µM); n=3/group. Results are expressed as mean ± SEM. *p < 0.05.

We then examined whether a relationship could exist between the increase of ECS tone observed in the WAT of HFD mice and the concomitant decrease in lipolysis. Since the assay medium used to measure lipolytic activity was deprived in insulin, regulation due to an alteration of insulin-sensitivity should be excluded. On the other hand, pathways regulating cAMP production might be involved as CB1R hyperactivation could lead to a decrease in cAMP production in HFD WAT. To test this hypothesis, we exposed epidydimal explants to the CB1R blockers Rimonabant and JD5037 during norepinephrine stimulation and measured glycerol release. While a stimulating action of Rimonabant existed in WAT explants from lean mice (**Figure S3**), we observed that CB1R blockade had no apparent effect on norepinephrine-stimulated lipolysis in these *ex-vivo* conditions (**Figure 4A**). Interestingly, the same conclusion was drawn from experiments performed with both VAT and SAT explants collected from obese subjects and treated with Rimonabant (**Figure 4B**). In addition, while norepinephrine and insulin did modulate cellular cAMP levels in epididymal explants of obese mice as expected, treatment with the CB1R inverse-agonist Rimonabant which is known to stimulate adenylate cyclase, did not potentiate the effect of norepinephrine (**Figure 4C**). Furthermore, while AEA was able to decrease cAMP levels after stimulation of adenylate cyclase by forskolin in lean WAT (**Figure 2B**), Rimonabant did not modify this pathway in obese WAT (**Figure 4D**). In line with this, the stimulating effect of AEA on Akt phosphorylation reported in WAT explants from lean mice (**Figure 2C**) was abrogated in WAT explants from insulin-resistant mice (**Figure 4E**).

**Figure 5:**
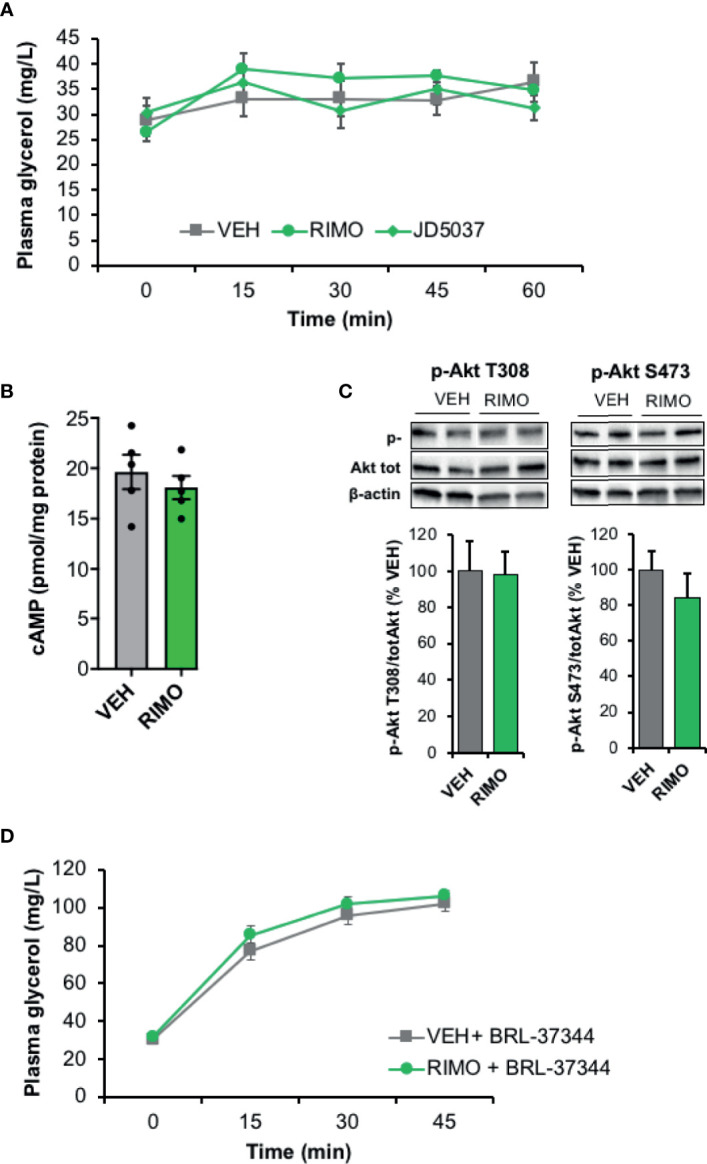
Blockade of WAT CB1R does not modify lipolysis in obese mice *in vivo*. **(A)** Kinetic of plasma glycerol (0, 30, 45 and 60 min) in mice fed with a high fat diet (HFD) after a single intraperitoneal injection of vehicle (VEH), Rimonabant (RIMO, 10 mg/kg) or JD5037 (3 mg/kg); n=9/group. **(B)** Intracellular cAMP content in WAT collected from HFD mice 45 min after an intraperitoneal injection of VEH or RIMO (10 mg/kg); n=4/group. **(C)** Representative immunoblots and densitometry analyses of the phosphorylation level of Akt (T308, S473) in WAT collected from HFD mice 15 min after an intraperitoneal injection of VEH or RIMO (10 mg/kg); n=5/group. **(D)** Kinetic of plasma glycerol (0, 30, and 45 min) in HFD mice after an intraperitoneal injection of BRL-37344 (5 mg/kg) in the presence of vehicle (VEH) or Rimonabant (RIMO, 10 mg/kg); n=4/group. Results are expressed as mean ± SEM.

When the impact of CB1R blockade on lipolysis was tested *in vivo*, acute administration of Rimonabant or JD5037 to HFD mice did not significantly modify basal plasma glycerol production (**Figure 5A**). In accordance with the *in vitro* data, Rimonabant injection to HFD mice did not cause any change in cAMP WAT content (**Figure 5B**). These results were unexpected since CB1R inverse agonists are known to increase cAMP levels and could increase lipolysis by this way. Besides, Akt activity in WAT was also not modified by the injection of Rimonabant indicating that insulin-dependent mechanisms were not affected by the CB1R blocker in HFD-treated mice (**Figure 5C**). We also noticed that Rimonabant had no effect on lipolysis when stimulated with BRL-37344, a selective activator of β3-adrenergic receptors (**Figure 5D**) suggesting that related regulating pathways were not modified by CB1R in our conditions. Finally, these *in vivo* and *in vitro* results obtained from obese WAT, collectively suggest that in the context of obesity, CB1R do not interfere with lipolysis as it does in control WAT.

## Discussion

In addition to play an important role in maintaining whole body energy homeostasis, WAT also helps to prevent ectopic lipid deposition and lipotoxicity due to its unique lipid storage capacity (29, 30). In the postprandial state, high plasma insulin levels typically stimulate fatty acid storage and suppress intracellular lipolysis (31). Conversely, when mobilization of endogenous energy stores is required in conditions like prolonged fasting or exercise, NEFA release is promoted by the combined effects of reduced plasma insulin and increased production of catecholamines (32).

It has been proposed that ECs and CB1R are essential mediators for functions aimed at accumulating energy for future use (1). In line with this, our investigation is the first one to demonstrate, using *in vivo* and *in vitro* strategies, that an acute elevation of ECS tone in WAT induces a rapid decrease in fasting lipolysis that could result from the activation of insulin signaling pathway. These findings join with and add to others indicating ECS activation promotes fat accumulation enhancing fatty acid uptake, lipogenesis and cell differentiation (18). Of note, parallel experiments performed on VAT and SAT explants collected from healthy subjects indicate that the inhibition of lipolysis by AEA also applied to human for both visceral and subcutaneous fat depots.

Evidence exists for an effect of ECs on norepinephrine release by sympathetic terminals innervating WAT (33). In line with this, the decrease in plasma glycerol contents observed in JZL195-treated or HFD mice could be the consequence of the backward action of ECs produced in excess by WAT on presynaptic CB1R which would lead to the inhibition of norepinephrine release as previously suggested (34). However, this possibility should be excluded in our study since the variations in lipolysis were maintained in experiments with tissue explants in the presence of exogenous norepinephrine.

Data obtained from experiments performed with FSK (**Figure 2B**) also suggest that the reduction in lipolysis may also be consecutive to a decreased in cAMP cellular levels since CB1R is negatively coupled to adenylyl cyclase through Gi/o signaling (35).

However, our findings support the view of the existence of another mechanism by which ECS may limit fat mobilization through the stimulation of the PI3K/Akt signaling pathway leading to an increase in cAMP degradation and finally to an inhibition of lipolysis. The existence of a crosstalk between ECS and insulin signaling pathways has been reported in different tissues while the potential mechanisms involved remain unclear. Indeed, it has been shown that acute or long-term modulation of CB1R activity affected insulin signaling through different mechanisms and induced sometimes divergent effects (36–39). In line with this, we reported in a previous work (22) that acute stimulation of CB1R impaired insulin-induced Akt phosphorylation in adipose tissue whereas Akt-pathway was activated in the present experiments performed in absence of insulin. In agreement with our finding, it has been clearly demonstrated that activation of CB1R produced a robust stimulation of Akt in Chinese hamster ovary cells transfected with CB1R in insulin-free conditions (40).

In addition to promote fat storage, a decrease in lipolysis is also predicted to decrease ectopic TG deposit and protect from the development of metabolic diseases (41). However, as the capacity of WAT to expand is limited, excessive lipid accumulation progressively induces adipose tissue hypertrophy favoring adipocyte dysfunction and finally impact the whole metabolism (42, 43). Taken together, our data support the concept that activation of ECS in WAT may progressively contribute to fat remodeling and tissue dysfunction limiting fat mobilization.

Dysfunctional WAT in obesity is characterized by an increase in basal lipolysis and an impairment of fat mobilization (44, 45). More specifically, the stimulating effect of catecholamines on lipolysis is reduced in SAT but increased in VAT (25). Besides, ECS tone has been positively correlated to fat mass and associated with metabolic risk both in human and mice (12, 46). Different studies have shown that WAT ECS is dysregulated by HFD feeding and associated with alterations of ECs tissue and plasma concentration (47, 48). However, data specifically dealing with the production rate of ECs by hypertrophied WAT are lacking. Our results provide direct evidence that epididymal WAT (referred to as VAT) from insulin-resistant mice secretes more AEA than lean mice. Since we observed a concomitant reduction in plasma glycerol production in HFD mice, we hypothesized that over-produced ECs may inhibit lipolysis activating local CB1R. Because the CB1R inverse-agonist Rimonabant is known to increase cAMP (49), it was expected to normalize or stimulate fat mobilization in HFD WAT. In direct line with this, two well-illustrated and elegant recent studies demonstrated the stimulating effect of CB1R blockade on lipolysis in adipocytes from lean rats (50) and in 3T3 cells (51). In the present work, while the action of Rimonabant also existed in lean WAT explants, no effect of the drug was observed on lipolysis in WAT explants from HFD mice. These findings are in accordance with another study in which the authors failed to show a direct action of Rimonabant on lipolysis at the tissue level in obese rats (52).

It is unclear why acute CB1R blockade neither modified cAMP levels nor Akt activity in obese adipose tissue. However, one explanation could be that the crosstalk existing between CB1R and Akt-pathway is ineffective in hypertrophied WAT in which the whole insulin-signaling pathway is altered (53). It could also be speculated that an alteration of some G-protein receptor-coupled mechanisms occurs in pathological adipocytes leading to an impairment of CB1R-mediated transduction signals. Indeed, in obese WAT, long-term exposure of CB1R to ECs may induce cellular adaptations such as CB1R desensitization as already described in brain (54).

Many studies have reported that CB1R blockade with Rimonabant or other peripheral-restricted antagonists increased energy expenditure and decreased fat mass (55). However, these conclusions mostly resulted from chronic pharmacological treatments that may induce long-term adaptations in different organs and thus indirectly improve WAT metabolism including fat mobilization. The major role of WAT CB1R in regulating energy balance has also been illustrated in a model of transgenic mouse with selective and inducible CB1R deletion in adipocytes. Indeed, the triggering of CB1R deletion in mice with established obesity induced body weight reduction and metabolism improvement (7). In their study, the authors placed CB1R at the center a crosstalk between adipocytes, immune cells and the sympathetic nervous system affecting WAT inflammation and adipocyte reprogramming. Thus, even if we did not show any effect of acute CB1R antagonism, one could speculate that peripheral CB1R blockers exert their beneficial effects over the long-term stimulating adipocyte browning as already observed in cell cultures (56) and counteracting the action of overproduced ECs on tissue inflammatory status targeting macrophages which represent a substantial proportion in the WAT of obese subjects (57). Further studies should be conducted to investigate the effects of ECs produced by expanding WAT on adipocyte reprogramming and on the secretion of pro-inflammatory cytokines from both adipocytes and non-adipocyte fraction.

**Figure 6:**
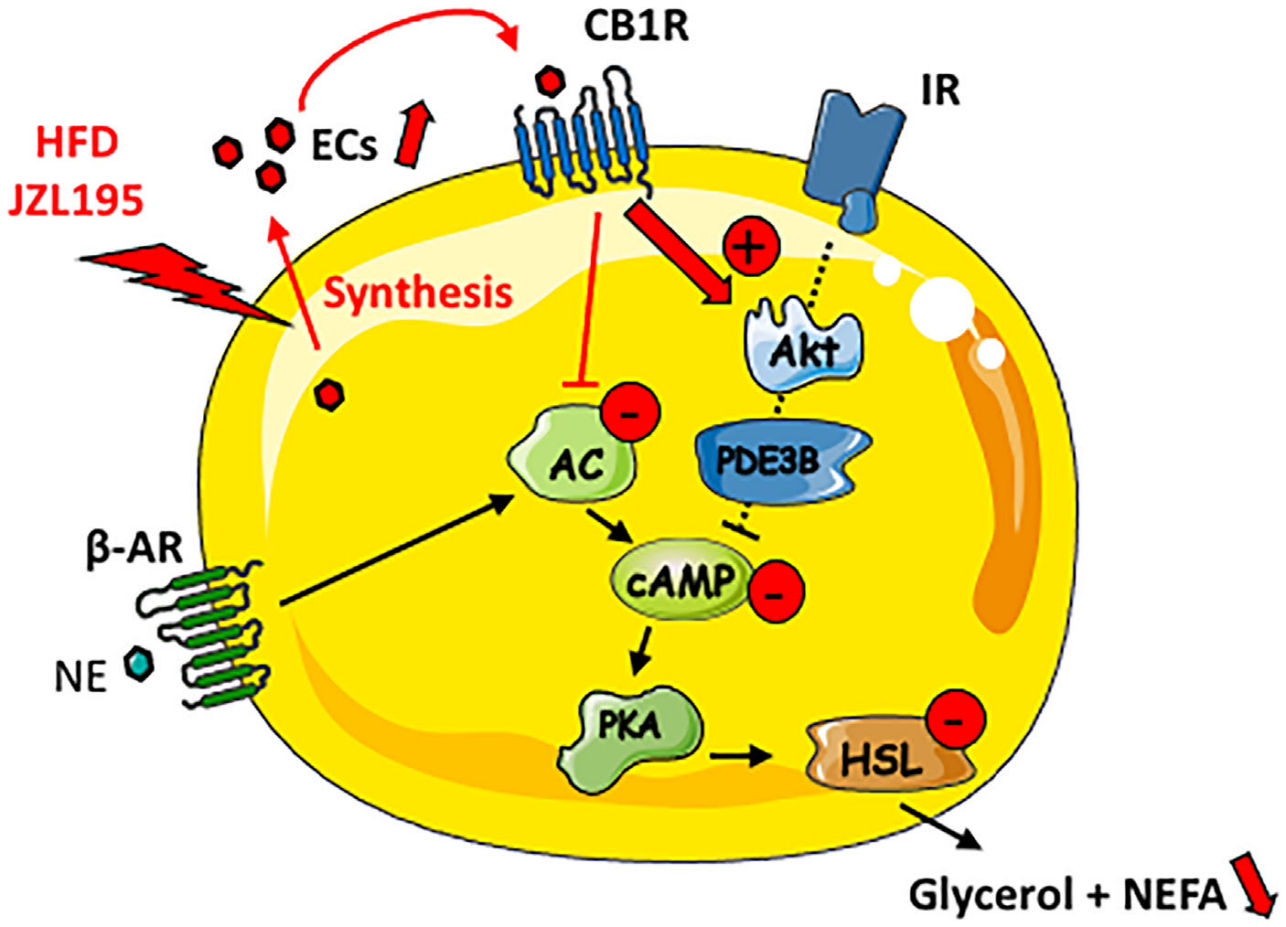
Putative schematic representation for the cAMP-dependent lipolysis regulation by locally produced ECs in lean WAT. AC, adenylate cyclase; ß-AR, beta-adrenergic receptor; IR, insulin receptor; NE, norepinephrine.

In conclusion, our study suggests that ECs secreted by WAT adipose exert an autocrine action on CB1R leading to the stimulation of PI3K/Akt signaling pathway which in turn limits fat mobilization (**Figure 6**). Thus, reducing WAT ECs production using pharmacological or nutritional strategies could help to prevent metabolic dysregulations link to fat mass expansion. The present findings also indicate that acute CB1R blockade is inefficient in regulating lipolysis in obese WAT, suggesting that the well-known beneficial effects of peripheral CB1R antagonists on WAT remodeling depend on other indirect and long-term mechanisms.

## Author’s Note

Parts of this work have been presented at the 29^th^ International Cannabinoid Research Society symposium (ICRS), July 2019, Bethesda, MD.

## Data Availability Statement 

The original contributions presented in the study are included in the article/**Supplementary Material**. Further inquiries can be directed to the corresponding author.

## Ethics Statement 

The studies involving human participants were reviewed and approved by French Committee for the Protection of Persons (CPP) (https://www.clinicaltrials.gov. Unique identifier: NCT03202706). The patients/participants provided their written informed consent to participate in this study. The animal study was reviewed and approved by Local ethics committee (CE2A, Dijon, France) for animal experimentation (APAFIS n°13948).

## Author Contributions 

CB, TM, LD, PP-D and PD designed, performed and analyzed the experiments. CB, TM, LD, JL, and PD wrote the manuscript. J-PP determined tissue endocannabinoid contents. POD organized human sample collection. TJ and BV supervised the experiments and critically revised the manuscript. All authors contributed to the article and approved the submitted version.

## Funding

This work was supported by INSERM, University of Burgundy and Franche-Comté and by a French government grant managed by the French National Research Agency (ANR) under the program Investissements d’Avenir with the reference ANR-11-LABX-0021-01-LipSTIC LabEx.

## Conflict of Interest

The authors declare that the research was conducted in the absence of any commercial or financial relationships that could be construed as a potential conflict of interest.

## Publisher’s Note

All claims expressed in this article are solely those of the authors and do not necessarily represent those of their affiliated organizations, or those of the publisher, the editors and the reviewers. Any product that may be evaluated in this article, or claim that may be made by its manufacturer, is not guaranteed or endorsed by the publisher.
