# Supplementary figures and images for: Endocannabinoids Produced by White Adipose Tissue Modulate Lipolysis in Lean but Not in Obese Rodent and Human
Source: Front Endocrinol (Lausanne). 2021 Aug 9;12:716431. doi: 10.3389/fendo.2021.716431 (PMC8382141; doi:10.3389/fendo.2021.716431)

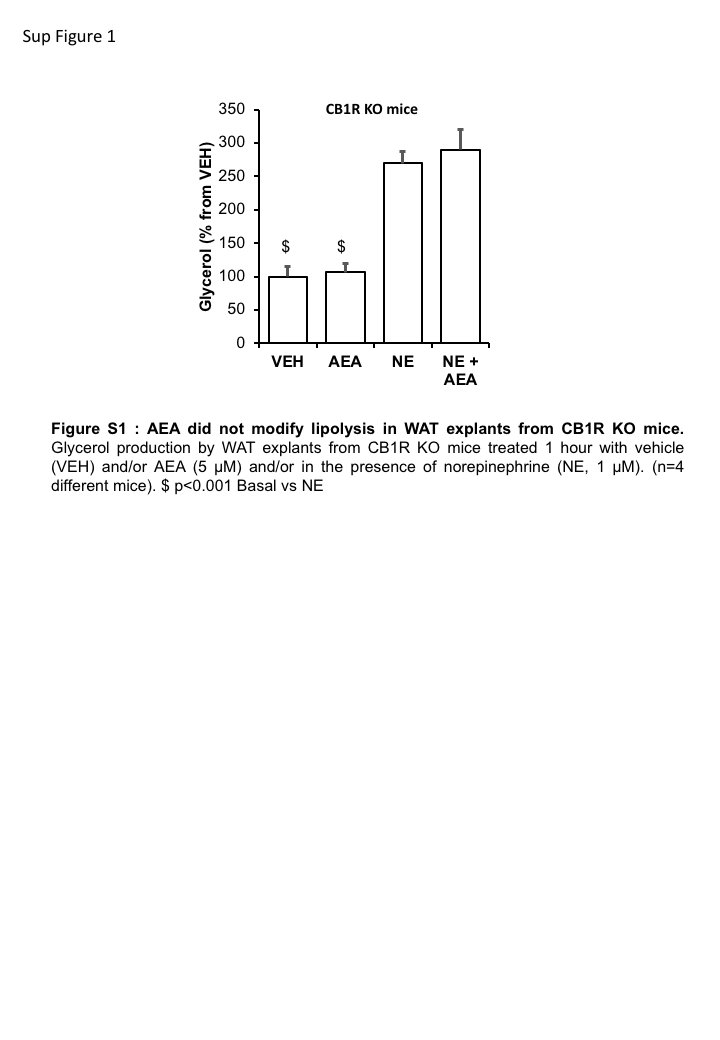

Supplement: Supplementary file 1 [file Image_1.tiff]

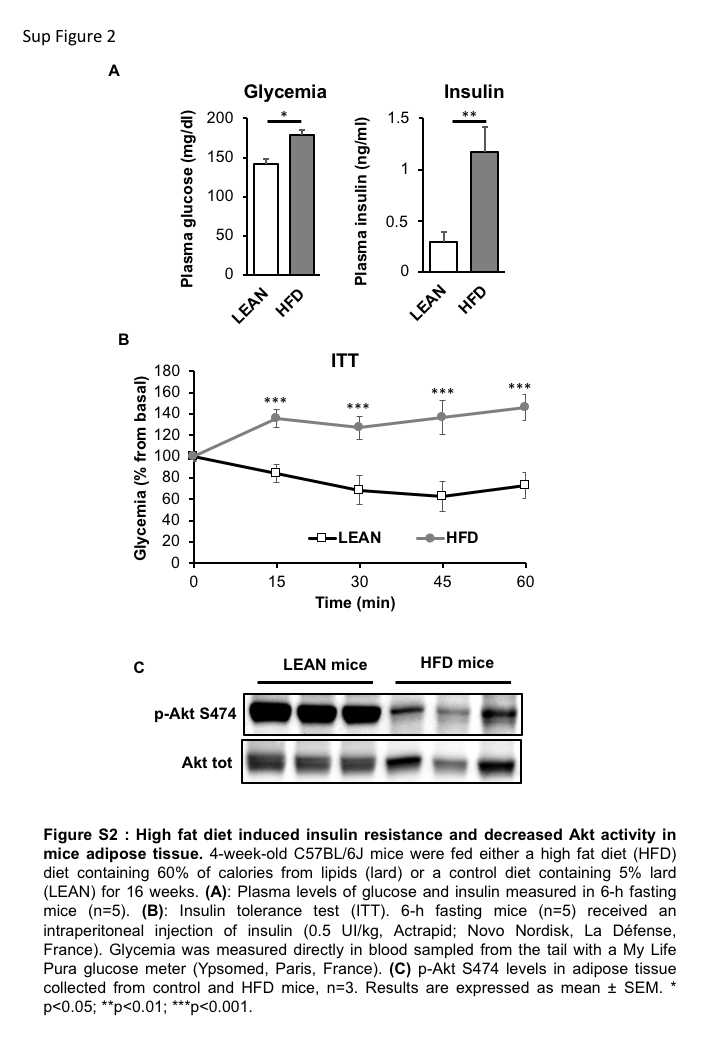

Supplement: Supplementary file 2 [file Image_2.tiff]

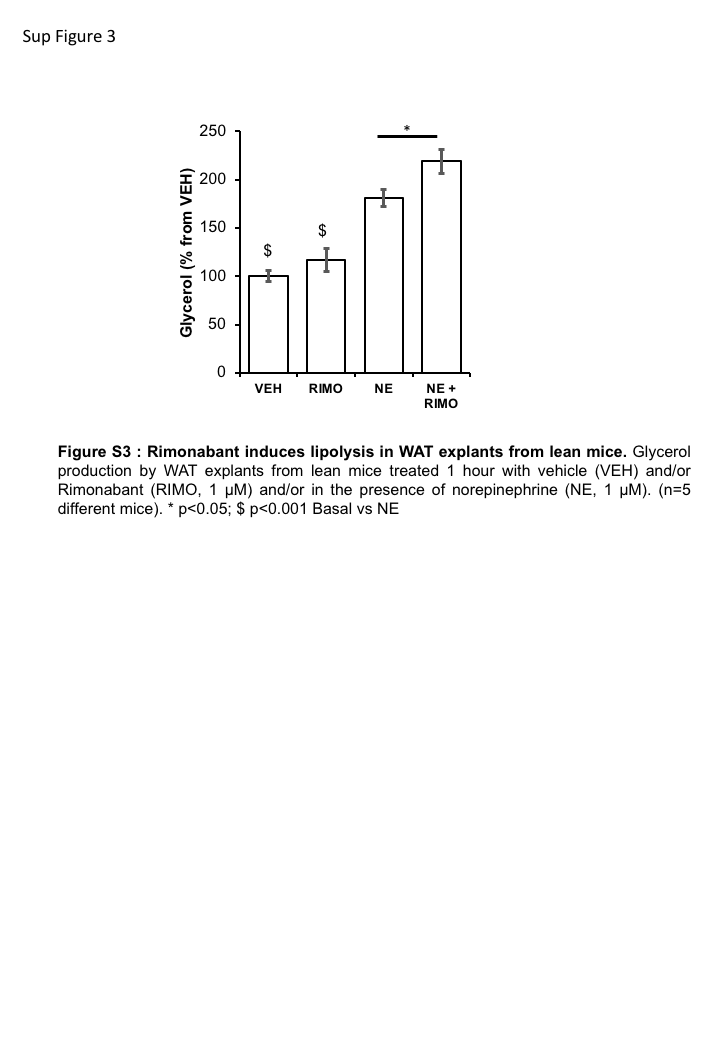

Supplement: Supplementary file 3 [file Image_3.tiff]
