# Supplementary material for: Endocannabinoids Produced by White Adipose Tissue Modulate Lipolysis in Lean but Not in Obese Rodent and Human
Source: Front Endocrinol (Lausanne). 2021 Aug 9;12:716431. doi: 10.3389/fendo.2021.716431 (PMC8382141; doi:10.3389/fendo.2021.716431)
Supplement: Supplementary file 4 [file Table_1.docx]

|  | | | |
| --- | --- | --- | --- |
| Name | **Species** | **Working dilution** | **Reference** |
| Phospho-Akt (Thr308) (C31E5E) | Rabbit | 1/1 000 | Cell signaling Technology, #2965 |
| Phospho-Akt (Ser473) (D9E) | Rabbit | 1/1 000 | Cell signaling Technology, #4060 |
| Phospho-Akt2 (Ser474) (D3H2) | Rabbit | 1/1 000 | Cell signaling Technology, #8599 |
| Akt (pan) (C67E7) | Rabbit | 1/1 000 | Cell signaling Technology, #4691 |
| Phospho-HSL (Ser660) | Rabbit | 1/1 000 | Cell signaling Technology, #4126 |
| HSL | Rabbit | 1/1 000 | Cell signaling Technology, #4107 |
| Phospho-PKA C (Thr197) | Rabbit | 1/1 000 | Cell signaling Technology, #4781 |
| PKA C-alpha | Rabbit | 1/1 000 | Cell signaling Technology, #4782 |
| Anti-Actin, clone C4 | Mouse | 1/ 1000 | Merck Milipore, MAB1501 |
| Anti-rabbit IgG, HRP-linked | Goat | 1/5 000 | Cell signaling Technology, #7074 |
| Anti-mouse IgG, HRP-linked | Horse | 1/5 000 | Cell signaling Technology, #7076 |
|  |  |  |  |

**Supplementary table 1 – List of antibodies used for western blotting**. All antibodies were diluted in 5% BSA-TBST.
