# Supplementary material for: Endocannabinoids Produced by White Adipose Tissue Modulate Lipolysis in Lean but Not in Obese Rodent and Human
Source: Front Endocrinol (Lausanne). 2021 Aug 9;12:716431. doi: 10.3389/fendo.2021.716431 (PMC8382141; doi:10.3389/fendo.2021.716431)
Supplement: Supplementary file 5 [file Table_2.docx]

|  | | | |
| --- | --- | --- | --- |
| Target | **Accession #** | **Forward (5’-3’)** | **Reverse (5’-3’)** |
| CB1R | NM_007726.3 | ccgcaaagatagtcccaatg | aaccccacccagtttgaac |
| NAPEPLD | NM_001359963.1 | agacgctggagtgagagagg | gctcgtccatttccaccat |
| FAAH | NM_010173.5 | ggaccttgctcccctttc | cctgctgggctgtcacata |
| DAGLa | NM_198114.2 | gctggacgaggcaaacaatg | ctcgtgcgagttcttgaggt |
| MAGL | NM_001166249.1 | cgcgaggtttccttccctaa | aaagtcaccccgattctccg |
| TBP | NM_013684.3 | acggcacaggacttactcca | gctgtctttgttgctcttccaa |
| L38 | AB037665.1 | catgcctcggaaaattgag | tcttgacagacttggcatcct |
| ATP5e | NM_025983.3 | tggactcagctacatccggt | aactcggtcttcagggcatc |

**Supplementary table 2 – List of primers used for real time RT-PCR**. Primer pairs were designed using NCBI/ Primer-BLAST and were synthesized by Eurofins Genomics France SAS (Nantes, France). CB1R: Cannabinoid 1 receptor; NAPE-PLD: N-acyl-phosphatidylethanolamine-hydrolyzing phospholipase D; FAAH: fatty acid amide hydrolase; DAGLa: Diacylglycerol lipase alpha; MAGL: Monoacylglycerol lipase; TBP: TATA box binding protein; L38: ribosomal protein L38; ATP5e: ATP synthase, H+ transporting, mitochondrial F1 complex, epsilon subunit.
